# Genome-wide association studies and genomic prediction for key ornamental traits in tetraploid pot carnation

**DOI:** 10.1186/s12870-026-10279-1

**Published:** 2026-10-27

**Authors:** Hugo H. Tavera, Dominik Losert, Maike Boxriker, Robert Boehm, Lydia Kienbaum, Sonali Dutta, Carola Zenke-Philippi

**Affiliations:** 1 Institute of Agronomy and Plant Breeding II, Justus Liebig University, Giessen, Germany; 2 Klemm + Sohn GmbH & Co KG, Stuttgart, Germany

**Keywords:** Tetraploid carnation, GWAS, Genomic selection, Plant-architecture, Flowering

## Abstract

**Background:**

Carnation (*Dianthus caryophyllus* L.) is one of the most commercially important ornamental crops worldwide. Similar to many ornamental species, genomic selection has yet to be widely adopted as a breeding tool in carnation. Moreover, the widespread development and commercialization of polyploid carnation cultivars makes their genomic study particularly relevant. In this study, we investigate the genetic architecture of key ornamental traits in a tetraploid pot carnation diversity panel using genome-wide association studies (GWAS), and evaluate the potential of genomic prediction for ornamental trait improvement in this panel. For genomic prediction, we assessed three SNP marker encodings capturing different allele dosage information, a pseudo-diploid encoding, a tetraploid additive dosage encoding, and an encoding accounting for both additive and non-additive genetic effects. In addition, we carried out a GWAS-assisted genomic prediction by fitting top-scoring GWAS markers as fixed effects to the genomic prediction model.

**Results:**

Through GWAS, we identified potential genomic regions of interest associated with seven of the eight traits studied. Genomic prediction achieved moderate to high prediction accuracy across traits and encodings, ranging on average from 0.54 to 0.82 for flowering-related traits (earliness, flower filling, flower count, and flower size) and from 0.39 to 0.48 for plant architecture traits (branching, habit, plant height, and vigour). Fitting top-scoring GWAS markers as fixed effects provided only modest gains in prediction accuracy (up to 0.03) for three of the eight traits (earliness, flower filling, and branching), with no improvement for the remaining traits under any of the three encodings.

**Conclusions:**

These findings extend the knowledge on the genetic basis of key ornamental traits and attest to the potential of genomic selection for breeding tetraploid pot carnation.

**Supplementary Information:**

The online version contains supplementary material available at https://doi.org/10.1186/s12870-026-10279-1.

## Background

Carnation (*Dianthus caryophyllus* L.) is a globally important ornamental species with high economic value [1]. Due to its morphological plasticity, carnation is not only relevant as a cut flower culture, but is also extensively used in landscaping and as pot plants. The diverse end-uses of carnation make the study of plant architecture and flowering related traits a priority in carnation breeding programmes [2]. However, as is the case for most ornamental species, breeding efforts in carnation still rely mainly on morphological markers and phenotypic selection, which is often time and resource intensive [3]. Thus, the implementation of genetic analyses has the potential to improve genetic gain in carnation.

Genomic selection has been shown to increase genetic gain in many plant species [4–6]. A key aspect of genomic selection is its potential to improve the breeding of traits with a complex genetic architecture and low heritability, for which traditional breeding approaches are often less effective [7]. In perennials, which include many commercially important ornamental species, genomic selection can be particularly beneficial due to its potential to shorten generation intervals in breeding programs [8–9]. In chrysanthemum and carnation, studies on genomic prediction have reported a high prediction accuracy for relevant ornamental traits including plant height, flower size, branching, and vase life [10–12]. Moreover, these studies also showed that prediction accuracy could be further improved when implementing a GWAS-assisted genomic prediction approach, where QTL detected by an association study are fitted as fixed effects in the genomic prediction model [13]. Despite the advantages of genomic selection and the widespread availability of molecular markers from next-generation sequencing, genomic selection remains largely untested for many ornamental species.

In major ornamental crops including rose, chrysanthemum, and carnation, the breeding and commercialization of cultivars with various ploidy levels is widely practised. Polyploid varieties enhance phenotypic plasticity in ornamental crops, contributing to the wide range of plant and flower morphologies [14]. However, determining the inheritance mode of polyploid cultivars, which is essential to carry out genetic analyses, can be challenging [15–16]. In general, polyploids are classified as either allopolyploids or autopolyploids, distinguished primarily by their mode of inheritance. Allopolyploids exhibit disomic inheritance as a result of preferential pairing between homologous chromosomes during meiosis, whereas autopolyploids exhibit polysomic inheritance arising from random multivalent pairing [17]. However, it has been long recognized that intermediate states also exist, where partial preferential pairing among homeologues gives rise to inheritance patterns that are neither fully disomic nor polysomic [18–19]. Modern rose cultivars are a well-studied example of this, where extensive hybridization between distinct but closely related species has resulted in so-called segmental allopolyploidy, characterised by inheritance patterns that are predominantly, but not entirely, tetrasomic [20]. Similar to rose, many tetraploid carnation cultivars have been developed through hybridization among closely related *Dianthus* species, suggesting that carnation could also be exhibiting an intermediate inheritance pattern [21–22].

In recent years, software tools like GWASpoly [23], SHEsisPlus [24], and MultiGWAS [25] have been developed to conduct association studies on polyploids and study the genetic basis of their traits. Likewise, studies in allo- and autopolyploid species have shown genomic prediction could be beneficial for their breeding programs [26–29]. To date, no studies have investigated the potential of genomic prediction for ornamental traits in tetraploid carnation. Based on this, the objectives of this study were to (1) conduct genome-wide association studies to investigate the genetic basis of key ornamental traits in a tetraploid pot carnation diversity panel, (2) evaluate the potential of genomic prediction for these traits and compare how SNP marker encodings that capture different allele dosage and gene action effects affect prediction accuracy, and (3) assess the impact on prediction accuracy when the genomic prediction model is extended by fitting top-scoring SNP markers detected by GWAS as fixed effects.

## Methods

### Phenotypic data

Eight ornamental traits (earliness, flower filling, flower count, flower size, branching, habit, plant height, and vigour) were evaluated in a diversity panel of 293 tetraploid pot carnation cultivars in a multi-environmental trial conducted at two locations over two years. Field trial layouts were generated using the package SudokuPlantDesign.jl [30] with Julia version 1.12.6 [31], which uses a practical implementation of augmented quasi-sudoku designs for plant breeding experiments. Augmented quasi-sudoku designs optimise for a homogeneous spatial distribution of check plots, with test cultivars subsequently assigned at random to the remaining plots [32]. All cultivars were provided by Selecta One (Klemm + Sohn GmbH & Co. KG, Stuttgart, Germany). The multi-environment trial was conducted across two locations (Stuttgart, Germany, and Latina, Italy) and two consecutive growing seasons (2023 and 2024). In Stuttgart, the 2023 design used 2 × 9 blocks within a 32 × 18 plot grid, whereas the 2024 layout employed 1 × 12 blocks within a 16 × 24 grid. In Latina, the 2024 design used 2 × 6 blocks arranged within a 66 × 6 plot grid. In all cases, layouts were optimised under identical structural principles (rectangular plot grids, incomplete blocking, and a fixed number of checks) to ensure comparability across environments while maintaining randomised allocation within each location-year combination. As only exception, due to limited cuttings availability, the 2023 Italy trial followed a breeder-defined layout with manually assigned rows and columns and irregular block sizes, resulting in an unbalanced row-column structure and varying block sizes. The number of cultivars, check cultivars and their replication, plants per plot, and missing observations for each location-year combination are summarised in Additional file 1 (Table S1).

The statistical analysis of the multi-environment trial followed a two-stage approach. In the first stage, each environment (location x year combination) was analysed independently to account for local spatial heterogeneity. Spatial adjustment was performed using the SpATS (Spatial Analysis of Field Trials with Two-dimensional P-splines) mixed model framework implemented in the R package SpATS version 1.0–19 [33]. The trait of interest was used as the response variable, with genotype fitted as random effect to reflect the breeding context. Two-dimensional spatial variation was accounted as a P-spline surface, which decomposes spatial heterogeneity into directional trends. The layout blocking factors were included as additional random effects, and no fixed effects beyond the intercept were specified, allowing the P-spline surface to capture systematic spatial trends without imposing rigid parametric assumptions. Genotypic BLUPs (best linear unbiased predictions) from each single-environment model were then used in the second stage for multi-environment analysis. In the second stage, the combined dataset was analysed using a linear mixed model implemented in the R package lme4 version 2.0–1 [34]:

$$\begin{array}{cl}y_{jklm}=&\mu+location_{j}+year_{{k}}+(location\times{year})_{jk}\\&+genotype_{l}+({genotype}\times{location})_{lj}\\&+({genotype}\times{year})_{lk}+\epsilon_{jklm}\end{array}$$

where y is the phenotypic observation for the j-th location, k-th year, l-th genotype, and m-th replicate; µ is the overall mean; location (Stuttgart or Latina) and year (2023 or 2024) are the main effects of location and year, respectively; the location x year term is the interaction between location and year; genotype is the main effect of each genotype tested in the trial; the genotype x location and genotype x year terms are the genotype-by-environment interaction effects, and ε is the residual error. All terms except the overall mean were treated as random factors. The resulting genotypic BLUPs, accounting for environmental variation and genotype-by-environment interactions, were used as the final phenotypic values for GWAS and genomic prediction. Differences in the precision of first-stage estimates were not accounted for when combining the first-stage outputs into the second stage. A comparison of genotypic values obtained from first-stage BLUEs and BLUPs showed a correlation of 0.99 with no relevant change in genotype ranking, so this choice is expected to have a negligible effect on the GWAS and genomic prediction results. Singular-fit diagnostics on this model showed no identifiability issues. Data distribution was assessed visually through histograms. Correlation between traits was evaluated with Pearson’s correlation coefficient. Histograms and correlation plots were rendered with the R package ggplot2 version 4.0.1 [35].

The traits were evaluated as follows: earliness was documented as the number of days until half of the flower buds on a plant had opened. Flower filling, evaluated as density of petals per flower at full bloom, was scored on a nine-point scale from single flower with five petals (1) to double flower with the highest petal density (9). Flower count, evaluated as the number of open flowers per plant, was scored on a nine-point scale ranging from the plant with the lowest number of open flowers (1) to the plant with the highest number of open flowers (9). Flower size, defined as the diameter of the corolla at full bloom, was scored on a nine-point scale from the smallest (1) to the largest flowers (9). Branching, defined as the density of branches between the lowest node and the uppermost flower buds, was scored on a nine-point scale from the lowest branch density (1) to the highest branch density (9). Habit, defined as the growth direction of the plant, was scored on a nine-point scale from a prostrate (1) to an erect growth form (9). Plant height, measured from soil level to the top of the plant, was scored on a nine-point scale from the shortest (1) to the tallest plant (9). Vigour, defined as the combination of inter-node density and amount of foliage, was scored between very sparse (1) to very dense biomass (9). Trait evaluation and scoring was partially based on the Protocol for tests on distinctness, uniformity and stability for *Dianthus* L., from the European Union Community Plant Variety Office [36]. Branching, habit, and vigour were scored at the full vegetative stage shortly before flowering, flower filling, flower size, and plant height were scored at full flowering stage, and flower count was assessed and scored individually for each plant rather than at a standardized developmental stage. Representative images for the scoring range for each trait can be found in Additional file 1 (Fig. S4).

### Genotypic data

Genotyping of 293 cultivars from the tetraploid pot carnation diversity panel was carried out by LGC Genomics (Berlin, Germany) using a genotyping-by-sequencing (GBS) approach based on *Msl*I restriction-site reduced representation libraries, sequenced as 150 bp paired-end reads on Illumina NextSeq 500/550 v2 and NovaSeq 6000 platforms (Illumina, San Diego, CA), with an average yield of approximately 3 million reads per sample. Reads were demultiplexed with bcl2fastq v2.20, adapter- and quality-trimmed, and clustered de novo with CD-HIT-EST v4.6.1 [37] (maximum 5% sequence divergence, excluding singleton clusters and clusters supported by fewer than 500 reads). Quality-trimmed reads were aligned against the resulting cluster reference with Bowtie2 v2.2.3 [38], achieving a mean mapping rate of 91.3%. Variant discovery and tetraploid dosage genotyping were performed with Freebayes v1.2.0 [39] under a fixed ploidy setting of four, with a minimum base quality of 10, a minimum per-locus read coverage of 5, and genotype qualities reported for all calls; loci were additionally required to have a read count above 8 across samples.

From this genotyping pipeline, the obtained SNP markers were further filtered using the following criteria. SNP markers with more than two recorded alleles, more than 10% missing values and a minor allele frequency (MAF) below 10% were filtered out of the dataset. Individuals missing more than 10% of marker information were excluded from the analyses. After data filtering, 15,433 SNP markers and 287 individuals remained. Remaining missing genotype calls were imputed using k-nearest neighbors with the knncatimpute function of the R package scrime [40]. SNP marker sequences were aligned to the carnation reference genome from [41] to determine chromosomal positions. The alignment of the sequences was carried out with R package rBLAST version 1.6 [42]. Multiple matches for each SNP marker were filtered by maximizing the percentage of sequence identity, alignment length, and bitscore, as well as minimizing the number of gap openings, mismatches, and e-value. The filtered and mapped markers were used for all further analyses.

### GWAS

**Fig. 1:**
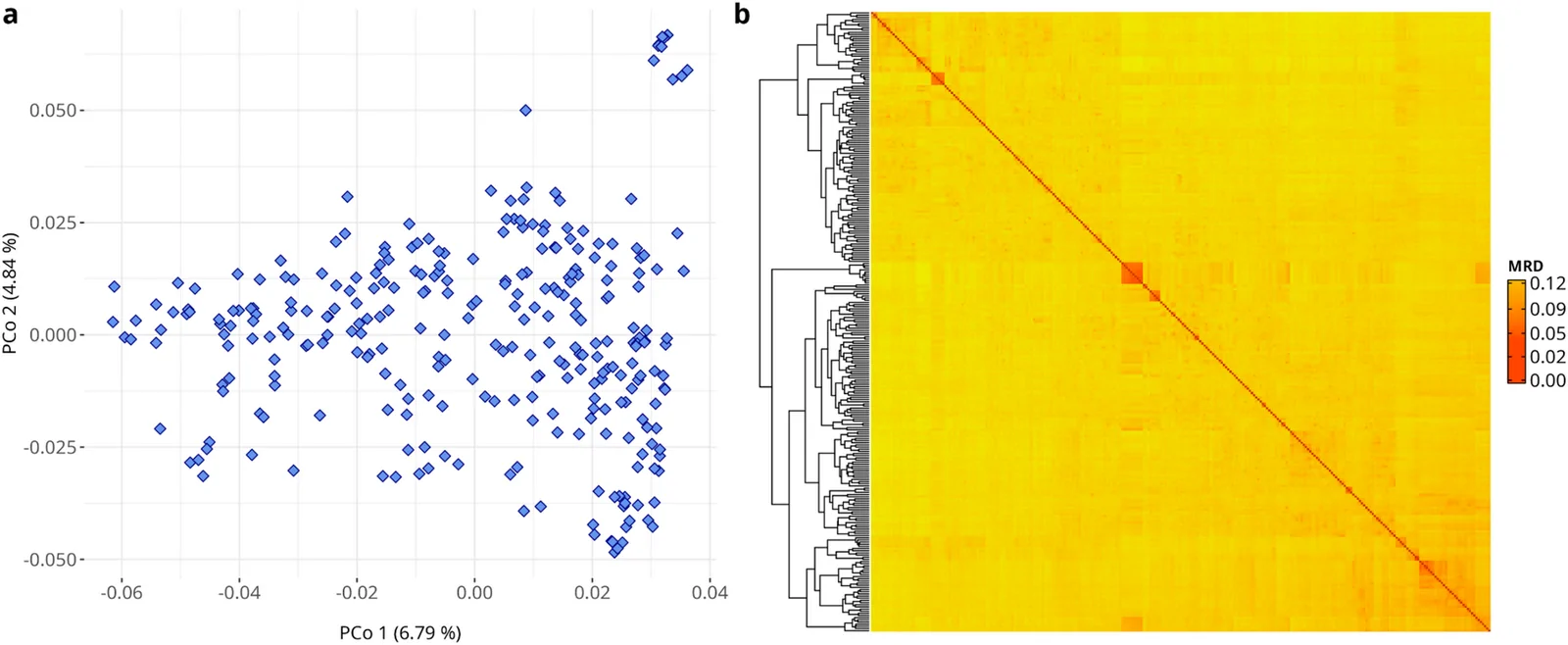
Population structure of the tetraploid pot carnation association panel (287 cultivars). (**a**) Principal coordinates analysis based on the pairwise modified Roger’s distances (MRD). Proportion of variance explained by the first (PCo 1) and second (PCo 2) principal coordinates. (**b**) Heatmap showing the distances between cultivars

Association between SNP markers and phenotypic data was conducted with the R package GWASPoly version 2.14 [23], which was designed to test for different genetic models in polyploid species. To control for population structure, the mixed linear model included the first principal component (Q) as a covariate and a kinship relationship matrix (K) calculated from all SNP markers available [43–44]. These settings were selected based on relatedness between individuals, visualized by a principal coordinates analysis plot and heatmap of genetic distances (Fig. 1), as well as suitability of the models observed on quantile-quantile (QQ) plots (Additional file 1, Fig. S1). SNP markers were evaluated under three gene action models: additive, simplex dominance (1-dom), and duplex dominance (2-dom) (Table 1). In the additive model, the effect of a SNP marker is proportional to the dosage of the minor allele. In the simplex dominance model, all three heterozygous states of a genotype are equivalent to one of the homozygous states, meaning that a single copy of the dominant allele is enough to completely mask the effect of the recessive allele. In the duplex dominance model, only the two heterozygous states with more than one copy of the dominant allele are equivalent to one of the homozygous states, meaning that at least two copies of the dominant allele are needed to mask the recessive allele. For the dominance models, both reference and alternate alleles were tested as dominant (dom-ref, dom-alt) [23]. To account for multiple testing, a significance threshold of *p* < 0.05 was set for each gene-action model with the Meff adjustment implemented in the set.threshold function of GWASPoly (method = “M.eff”), a Bonferroni-type correction using an effective number of markers estimated from pairwise marker correlations [45]. This correction was applied independently for each gene-action model parameterization tested by GWASPoly (additive, 1-dom-ref, 1-dom-alt, 2-dom-ref, 2-dom-alt). The number of effective markers, and the resulting threshold for each model, are reported in Additional file 1 (Supplementary Methods, Table S3).

Effects of the allele substitution on the phenotype were obtained as the regression coefficients of the respective gene-action model for SNP markers above the significance threshold. For the additive model, this coefficient represents the effect per copy of the alternate allele; for the dominance models, it instead represents a contrast between the two genotype classes coded 0 and 4 (Table 1) (e.g. nulliplex versus all other genotypes for the 1-dom-alt model), rather than a per-allele effect. The proportion of phenotypic variance explained by each significant SNP marker was estimated using the fit.QTL function of GWASPoly, as the partial R^2^ = 1-exp(-D/n), where D = 2(LLfull - LLreduced) is the deviance between a full model including all significant markers detected for that trait and a reduced model omitting the focal significant marker, both fitted by maximum likelihood, n is the number of individuals, and D is compared against a Chi-square distribution with degrees of freedom equal to the number of parameters of that marker’s gene-action model. When multiple significant markers were detected for the same trait, this R^2^ therefore reflects each marker’s conditional contribution given the others, rather than its marginal explanatory power alone.

**Table 1:** Encodings of design matrices for GWAS with GWASPoly for three gene action models. Based on [23]

Genotype	Additive	1-dom A > B	1-dom A < B	2-dom A > B	2-dom A < B
AAAA	0	0	0	0	0
AAAB	1	0	4	0	0
AABB	2	0	4	0	4
ABBB	3	0	4	4	4
BBBB	4	4	4	4	4

### Genomic prediction

Genomic prediction was carried out based on ridge regression best linear unbiased prediction (RR-BLUP) [46]. Three SNP marker encodings were considered for the design matrices of the genomic prediction model (Table 2). The pseudo-diploid (PD) encoding represents a diploidized allele dosage, where the three possible heterozygote states are reduced to one. The additive (A) encoding represents a full tetraploid allele dosage of additive effects, where the total SNP marker effect is proportional to the number of copies of the alternate allele. The additive and non-additive (AD) encoding assumes that each genotype has its own effect and there are five possible effects for each SNP marker [26]. For the baseline model of each encoding, the whole set of SNP markers (15,433) was fitted as random effects.

For the GWAS-assisted genomic prediction model, the SNP markers with highest scores (referred to as top-scoring SNP markers) were fitted as fixed effects into the baseline model. The top-scoring SNP markers were selected and sorted in descending order based on their -log10(p) score. The model was fit with 0 (baseline), 1, 2, 5, 10, 20, 50, 100, or 120 top-scoring SNP markers as fixed effects. For the PD and A encodings, this corresponds to one fixed-effect column per marker plus an intercept; for the AD encoding, the marker coding was reduced to four full-rank columns per marker (dropping the redundant nulliplex column as the reference level), giving 4n + 1 fixed-effect parameters for n markers (e.g. 201 parameters at *n* = 50). Marker counts of 100 and 120 were excluded for the AD encoding, as the resulting fixed effects matrix (401 and 481 parameters, respectively) would otherwise exceed the roughly 230 training individuals per fold. Additionally, the rank of the fixed effects matrix was checked for every cross-validation run and encoding, and any column corresponding to a genotype class absent from that training split was removed. Top-scoring SNP markers fitted as fixed effects were not removed from the random-effect marker set [47]. To evaluate the performance of the top-scoring SNP markers, the A encoding was additionally tested by fitting the same marker counts of randomly selected SNP markers as fixed effects. For each encoding scheme, the same encoding was used for both the fixed and random effects matrices of the model. The model was evaluated as:

$$y={Xb}+{Zu}+\epsilon$$

where y is a vector of genotypic BLUPs for a given trait, b is a vector of fixed effects for the top-scoring SNP markers fitted to the model, u is a vector of random effects and ε is a vector of residual errors. X and Z are design matrices relating b and u to the phenotypic records. Xb includes an intercept. The encoding of the SNP markers in X and Z changed depending on the encoding scheme, described in Table 2. We assumed distributions of u ∼ N(0, Iσ²u) and ε ∼ N(0, Iσ²ε), where σ²u and σ²ε are the genetic variance and the residual variance, respectively. I is an identity matrix.

The prediction accuracy of the fitted models was assessed through cross-validation. For each of 1,000 cross-validation runs, the dataset was randomly split as 80/20 into training and validation sets. A GWAS was re-run independently within the training partition of each cross-validation run to select the top-scoring SNP markers to include as fixed effects. Pearson’s correlation between observed phenotypic values y and predicted phenotypic values y-hat from the validation set was employed as the measurement of prediction accuracy r(y, y-hat). We used the R package rrBLUP version 4.6.3 [48] to run the genomic prediction models. All calculations, with the exception of field trial layout generation, were carried out using the statistical software R version 4.5.3 [49].

**Table 2:** Encodings of design matrices for genomic prediction of a tetraploid carnation diversity panel

Genotype	Pseudo-diploid (PD)	Additive (A)	AD:1	AD:2	AD:3	AD:4	AD:5
AAAA	0	0	1	0	0	0	0
AAAB	1	1	0	1	0	0	0
AABB	1	2	0	0	1	0	0
ABBB	1	3	0	0	0	1	0
BBBB	2	4	0	0	0	0	1

AD columns 1–5 give the number of effects per marker for the additive and non-additive encoding. Based on [26]

## Results

### Population structure and characterizing carnation ornamental traits

Principal coordinate analysis (PCoA) of the tetraploid carnation diversity panel revealed a largely unstructured population, with the first two principal coordinates explaining 6.79% and 4.84% of the total variation, respectively (Fig. 1a). The heatmap of genetic distances with hierarchical clustering confirmed the limited population structure, with generally high pairwise distances among accessions and no strongly defined subgroups (Fig. 1b).

**Fig. 2:**
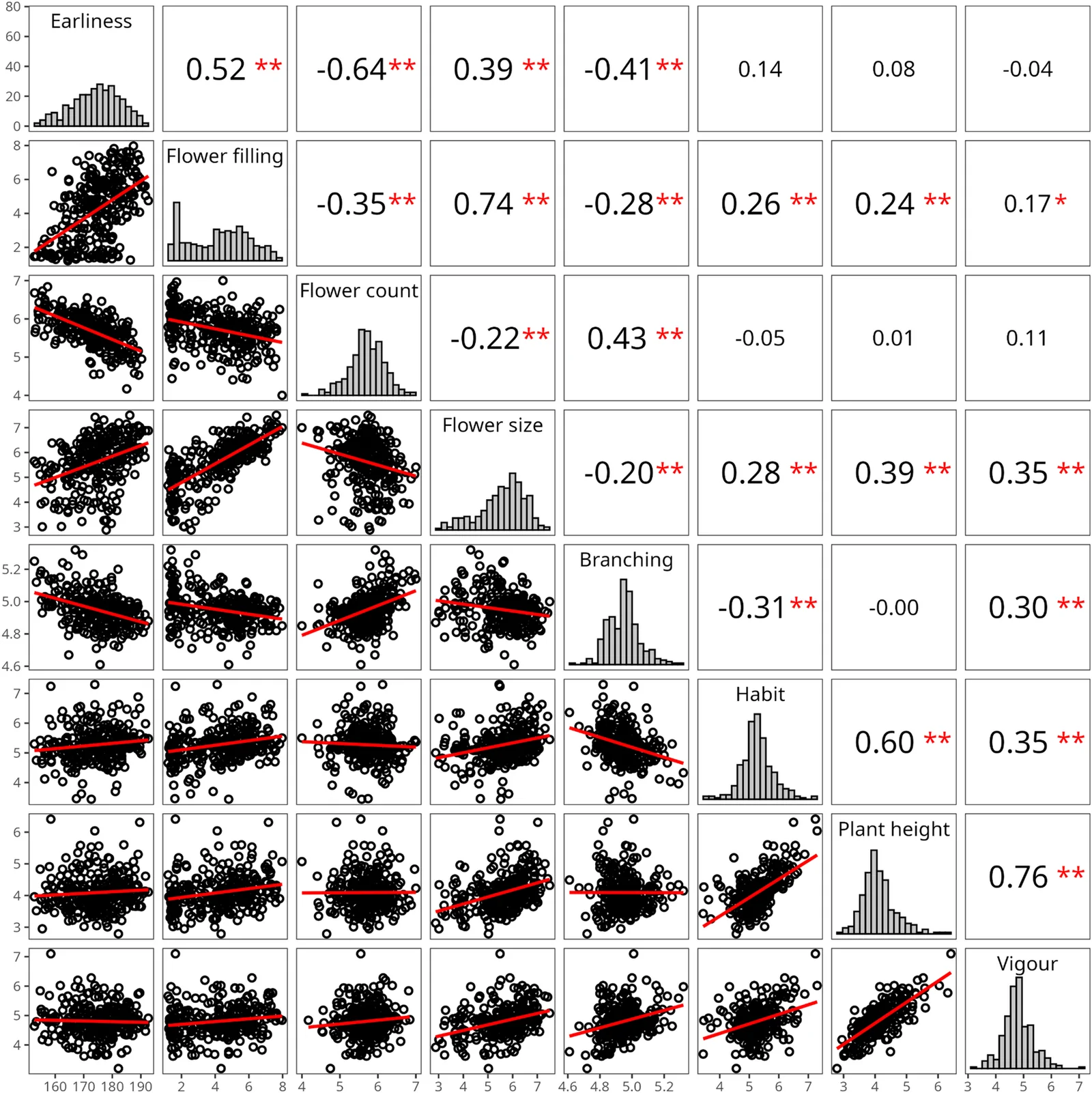
Correlation and distribution plots of the eight ornamental traits evaluated. Based on the genotypic BLUPs of a two-year two-location trial, one symbol and two symbols denote correlations significantly different from zero at *p* < 0.01 and *p* < 0.001, respectively

Phenotypic data from a two-location, two-year trial was collected for eight ornamental traits. Data for branching, flower filling, flower count, flower size, habit, plant height and vigour was collected as interval-scaled scores from 1 to 9. The trait earliness was measured in days. Histograms of the distribution from the genotypic BLUPs are presented in Fig. 2. All evaluated traits, except for flower filling, approximately follow a normal distribution. For flower filling, the phenotypic values were nevertheless not transformed, since the GWAS method employed is robust to slight deviations from normality.

Correlation coefficients were significant between most traits (Fig. 2). The strongest positive correlations were observed between vigour and plant height (*r* = 0.76, *p* < 0.001), followed by flower filling and flower size (*r* = 0.74, *p* < 0.001). Earliness and flower count had the strongest negative correlation (*r* = -0.64, *p* < 0.001). All traits showed a significant correlation to at least three other traits. Flower filling and flower size were both significantly correlated to all other seven traits.

### Genome-wide association studies

**Fig. 3:**
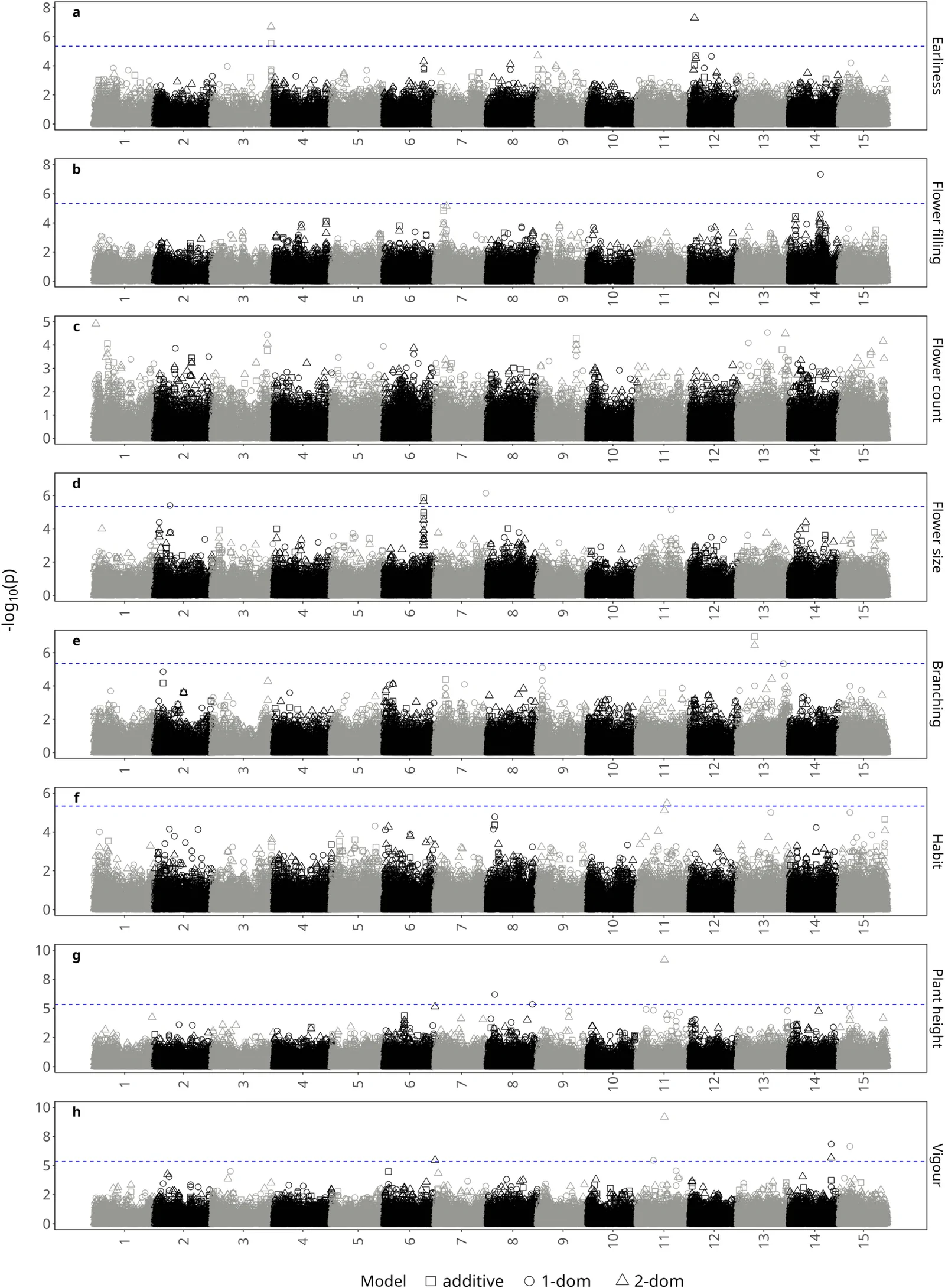
Manhattan plots of GWAS for eight ornamental traits in a tetraploid carnation diversity panel. Three gene action models were assessed for 287 cultivars and 15,433 SNP markers, additive (squares), simplex dominance (circles), and duplex dominance (triangles). Meff-adjusted significance threshold represented by blue dashed line (the most conservative of the five model-specific thresholds, -log10(p) = 5.34, shown where multiple models are overlaid in the same panel). (**a**) Earliness. (**b**) Flower filling. (**c**) Flower count. (**d**) Flower size. (**e**) Branching. (**f**) Habit. (**g**) Plant height. (**h**) Vigour

A GWAS was carried out for a tetraploid pot carnation diversity panel of 287 genotypes for eight ornamental traits. A total of 14,203 to 15,433 SNP markers were tested across the three gene action models, depending on the specific model parameterization. A significance threshold (*p* < 0.05) was set using the Meff adjustment, computed independently for each gene action model; resulting thresholds ranged narrowly from -log10(p) = 5.30 to 5.34 across the model parameterizations tested, and markers were evaluated against their own model-specific threshold (Additional file 1, Table S3). A total of 21 significant signals corresponding to 16 SNP markers were detected for seven out of the eight traits evaluated. The largest number of significant signals were detected for vigour, on chromosomes 6, 11, 14, and 15 (Fig. 3). The SNP marker 59,713 − 5658_179 exhibited the highest score across all traits. This SNP marker was detected both for vigour and plant height by the duplex dominance model. These were also the two traits with the highest correlation between each other (Fig. 2). Despite high correlation between most traits, no other SNP marker was detected for more than one trait. The second highest scoring signal was SNP marker 10,448–17081_103 located on chromosome 14. This was the only significant signal detected for flower filling. Four SNP markers were significant within the same trait for more than one gene action model: SNP marker 43,103 − 7244_187 for branching, 21,052 − 11671_135 for earliness, 8867–18575_6 for flower size, and 137881-2718_123 for vigour. Only for flower count no signals were observed above the significance threshold. A characterization of all significant signals is presented in Table 3. For all three models tested for each trait, the observed -log10(p) values closely followed the expected distribution across the low to middle range, suggesting proper control of false positive signals (QQ-plots, Additional file 1, Fig. S1).

**Table 3:** Characterization of significant SNP markers identified by GWAS

Marker	Chr	Pos(Mb)	MAF	Score	Effect	PVE(%)	Model	Trait
21,052 − 11671_135	3	41.92	0.13	5.56	-3.47	0.47	additive	earliness
21,052 − 11671_135	3	41.92	0.13	6.69	-7.54	3.55	2-dom	earliness
4082–26577_8	12	2.82	0.47	7.29	-5.16	9.22	2-dom	earliness
10,448–17081_103	14	21.92	0.37	7.33	1.16	8.85	1-dom	flower filling
109968-3369_74	2	10.85	0.16	5.40	-0.82	5.30	1-dom	flower size
8867–18575_6	6	28.07	0.19	5.85	-0.16	0.29	additive	flower size
8867–18575_6	6	28.07	0.19	5.64	-0.47	0.53	2-dom	flower size
82,073 − 4359_216	7	36.07	0.21	6.14	-0.91	7.23	1-dom	flower size
43,103 − 7244_187	13	12.06	0.12	6.98	-0.05	1.18	additive	branching
43,103 − 7244_187	13	12.06	0.12	6.44	-0.11	1.19	2-dom	branching
6687–21295_58	13	32.69	0.44	5.33	0.09	6.87	1-dom	branching
1331324-114_147	11	21.12	0.15	5.48	1.01	7.46	2-dom	habit
1914–36137_42	8	5.02	0.13	6.20	0.36	5.01	1-dom	plant height
50,045 − 6489_78	8	31.90	0.39	5.35	-0.50	4.69	1-dom	plant height
59,713 − 5658_179	11	19.29	0.11	9.17	2.57	10.02	2-dom	plant height
3609–28018_188	6	36.21	0.10	5.47	1.19	0.12	2-dom	vigour
1082510-165_61	11	11.34	0.32	5.44	-0.60	2.71	1-dom	vigour
59,713 − 5658_179	11	19.29	0.11	9.17	2.64	1.41	2-dom	vigour
137881-2718_123	14	29.59	0.10	6.84	0.94	0.44	1-dom	vigour
137881-2718_123	14	29.59	0.10	5.65	0.68	0.13	2-dom	vigour
809938-270_85	15	7.23	0.11	6.63	1.09	2.36	1-dom	vigour

MAF minor allele frequency; Score -log10(p); PVE phenotypic variation explained

### Genomic prediction

**Fig. 4:**
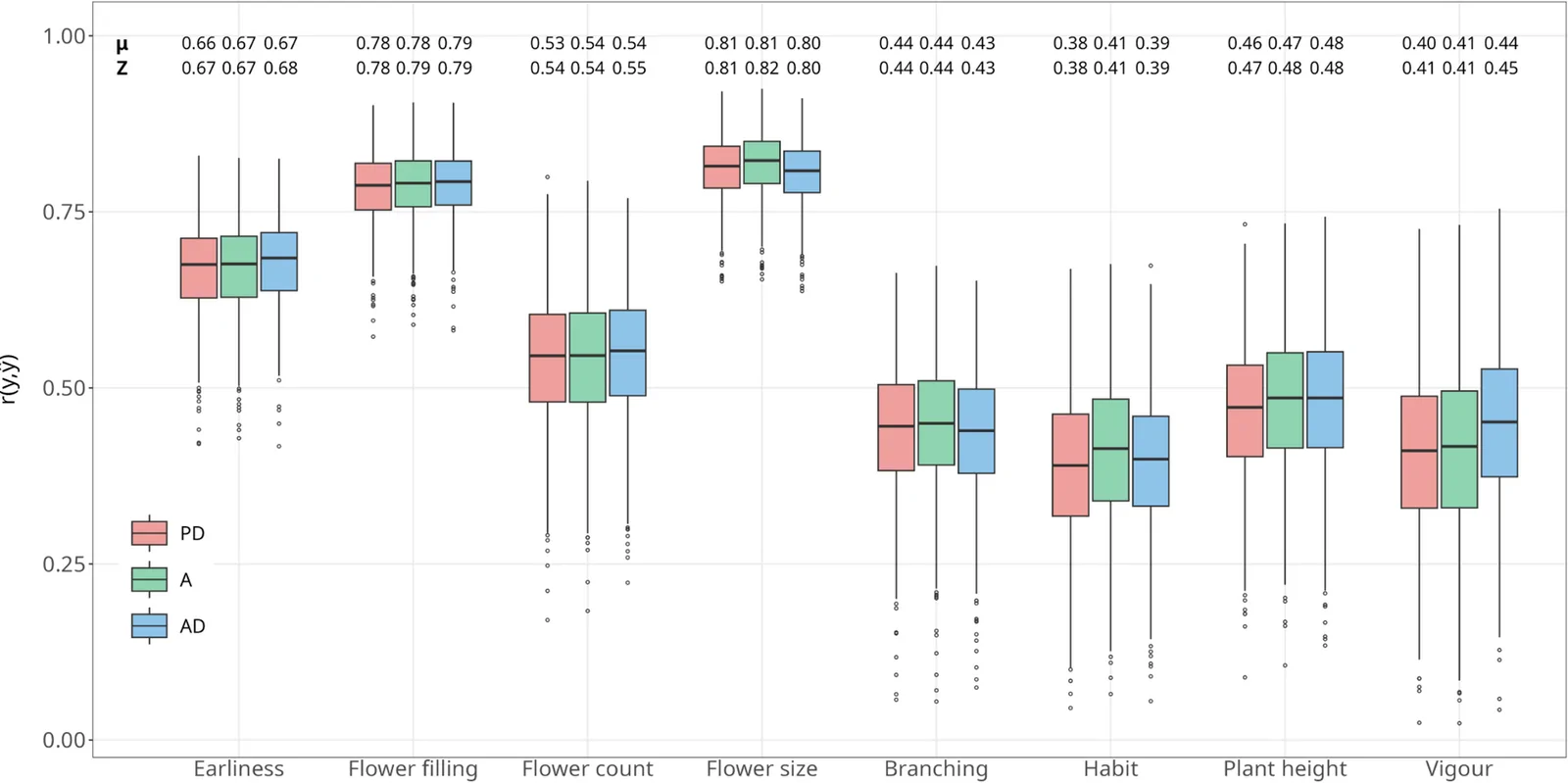
Genomic prediction of a tetraploid carnation diversity panel for eight ornamental traits with different SNP markers encodings. The evaluated encodings were pseudo-diploid (PD), additive only (A) and additive and non-additive effects (AD). The boxplots show the correlation r(y, y-hat) between the observed phenotypic values y and the predicted phenotypic values y-hat in the validation set for 1,000 cross-validation runs

Genomic prediction was conducted based on three SNP marker encoding schemes for eight ornamental traits. The encodings tested were pseudo-diploid (PD), additive effects only (A) and additive and non-additive effects (AD) (Table 2). Differences in prediction accuracy were minimal within each trait regardless of the SNP marker encoding employed (Fig. 4). Averaging over the three encodings, the flower morphology traits flower filling and flower size showed the highest average prediction accuracy (0.78 and 0.8, respectively). In contrast, habit exhibited the lowest average prediction accuracy (0.39). Prediction accuracies for all traits with the three encodings as well as mean and median values of the 1000 cross-validation runs are depicted in Fig. 4.

Fitting top-scoring SNP markers as fixed effects only marginally improved prediction accuracy over the baseline model for earliness, flower filling, and branching (Additional file 1, Fig. S3). For the remaining five traits, no number of top-scoring SNP markers outperformed the baseline model under any encoding. Where a gain was observed, the best-performing number of markers within this cross-validation design was consistently low (one or two), and neither the PD nor the A encoding showed a consistent advantage over the other. Increase in prediction accuracy with the AD encoding were smaller still, and restricted to the same three traits; marker counts of 100 and 120 could not be evaluated for this encoding, as the resulting fixed effects matrix would exceed the number of training individuals.

Fitting randomly selected SNP markers as fixed effects produced a similar pattern of declining accuracy with increasing marker number. For several traits, including earliness, prediction accuracy with random markers matched or exceeded that of the corresponding top-scoring SNP marker model at higher marker counts, indicating that the markers selected by GWAS carried little information beyond what would be expected by chance.

## Discussion

### Dissection of ornamental trait based on GWAS

Genome-wide characterization of genetic variants has been successfully applied to the study of quantitative traits, both on model and crop species over the last decades [50–51]. Similarly, in recent years, ornamental crops and their respective traits have been studied through QTL analyses and GWAS [52–54]. For carnation however, although belonging to the top economically important ornamental species worldwide, the genetic basis of many relevant traits remains to be formally investigated by locus-trait analyses [1]. Here, we implemented GWAS to investigate the genetic basis of eight key ornamental traits on a tetraploid pot carnation diversity panel. Our focus was primarily on conducting an exploratory analysis of their genetic architecture and draw comparisons to other species, rather than to identify the specific causal genes underlying the detected QTL.

Of the 16 SNP markers significantly associated with a trait, 10 belonged to plant architecture-related traits, namely vigour, plant height, habit and branching (Table 3). In ornamentals, these traits are of relevance due to their major role in aesthetic appeal and have been studied in key ornamental species like roses and chrysanthemum. In rose, vigour has been described as a complex trait and QTL analysis reported over 40 genomic regions associated with the trait [55–56]. Consistent with this, for vigour we identified the largest number of significant signals, which explained between 0.12 and 2.71% of phenotypic variation. Moreover, we observed a number of high scoring peaks under the significance threshold across seven chromosomes. Variation in this trait is influenced by numerous other plant architecture traits [57], reflected here in the significant positive correlations with branching, habit, and plant height. These results support a polygenic genetic architecture for vigour in carnation, where variation is controlled by many loci with small effects.

Plant height has been extensively studied in ornamental crops and is generally considered a quantitative trait. In rose, several genomic regions on chromosomes 1, 2, and 5 have been repeatedly associated with stem length, a key determinant of plant height [55, 58]. In chrysanthemum, GWAS identified multiple minor QTL for plant height distributed across nine chromosomes [10]. Here, although a limited number of SNP markers were significant for plant height, we observed a broad distribution of high scoring signals marginally below the significance threshold across 6 chromosomes. Furthermore, the strongest signal detected, explaining approximately 10% of phenotypic variation, was found on chromosome 11 for plant height, indicating a genomic region with a potential major effect on this trait, similar to reports for stem length in rose [58].

For branching, a GWAS in chrysanthemum identified a major QTL associated with a candidate region homologous to the phyB gene in Arabidopsis, which has been shown to regulate branching in response to light and shade conditions [59]. In sunflower (Helianthus annuus L.), branching has been shown to be largely controlled by a major QTL located on chromosome 10 [60]. Here, two SNP markers were detected for branching both located on chromosome 13. Additionally, two peak signals below the significance threshold were observed on chromosomes 2 and 9. Given that the phenotypic variation explained by the significant SNP markers was relatively small, it is unlikely that one of these represents a major QTL for branching, like reported for other species. Nevertheless, the different mechanisms underlying branching, such as primary branch length, number of lateral buds and branch angle, among others, appear to be controlled by a relatively small number of loci [59]. Dissecting these mechanisms individually would provide further insight into the genetic architecture of branching in pot carnation.

Although habit is a key trait in pot ornamental appeal, to our knowledge, no locus-trait analyses have been carried out for this trait in ornamental species. Association studies in other crops, including peanut, spring wheat, and rapeseed, have described habit as a complex trait [61–62]. Additionally, the range of growth habits exhibited by a species is strongly shaped by its domestication history, making habit a largely species-specific trait [61]. Here, we observed one significant SNP marker, explaining 7.46% of phenotypic variance. Moreover, high scoring signals below the significance threshold were observed on chromosomes 8, 13 and 15. The positive effect of the significant SNP marker detected suggests it contributes to more erect phenotypes. We speculate that within our diversity panel, the variation in habit forms is being reflected in the distribution of several high scoring signals across the genome, potentially indicative of a polygenic inheritance pattern for this trait. Notably, significant SNP markers for habit, plant height, and vigour were identified on chromosome 11 within the 11.34–21.12 Mb region, with the SNP marker 59,713 − 5658_179 detected for both plant height and vigour. These findings suggest that this genomic region may have a central role in shaping plant architecture in pot carnation, and that further research and fine-mapping of this region are warranted.

Flowering is a continuous and dynamic process that involves multiple developmental stages, including bud formation, early opening, and full bloom [52]. Moreover, certain carnation varieties exhibit recurrent blooming, meaning they can undergo flowering more than once, which further increases the complexity of the genetic mechanisms underlying this trait [63]. Flowering time, or earliness, is an important indicator of crop productivity and its genetic basis has been extensively studied for many major crops as well as ornamentals [64–66]. In rose, QTL analyses have identified multiple QTL with moderate to large effect sizes along several genomic regions for different rose varieties [56, 67–68]. Specifically for diploid carnation, a QTL analysis revealed a major QTL located on linkage group 10, which explained around 20% of phenotypic variation [52]. Here, both the additive and duplex dominance models detected the same SNP marker on chromosome 3, which was significantly associated with earliness and explained 0.47 and 3.55% of phenotypic variance, respectively. Furthermore, a second significant SNP marker was detected on chromosome 12 and explained 9.22% of phenotypic variance. Whether any of these signals correspond to the QTL reported for diploid carnation remains to be determined. A direct comparison of marker positions between studies is not straightforward, as in carnation, chromosome numbering and positional references are not standardised across independently constructed genetic maps. Nonetheless, genetic insights gained at the diploid level are frequently applicable to related polyploid species and sequence alignment of the markers could confirm whether both studies are tagging the same genomic region [58].

Flower morphology traits are equally important for commercial value in carnation. The genetic architecture of flower size and filling has been studied in rose and chrysanthemum. In rose, four QTL for flower size explaining between 36.1% and 47.9% of phenotypic variation were identified in two mapping populations on chromosomes 1 and 2 [67]. However, the authors argue that due to the small mapping population sizes, these values may be inflated and QTL with smaller effects might have been present but could not be detected. Specifically for petal size, a GWAS identified a single QTL explaining 10% of phenotypic variance in a panel of tetraploid rose [54]. In chrysanthemum, a GWAS identified five SNP markers significantly associated with capitulum diameter explaining between 27.6 and 31.2% of phenotypic variance. In this study, we observed three SNP markers on chromosomes 2, 6 and 7, explaining between 0.29 and 7.23% phenotypic variation, for flower size. Although the significant SNP marker on chromosome 6 explained the smallest proportion of phenotypic variation, several additional peak signals were observed in close proximity, indicating a potential region in high linkage disequilibrium associated with flower size. Flowers are composite structures made up of multiple specialized organs, including petals, sepals, stamens, and carpels, for which development is typically regulated by different sets of genes. As a result, overall flower size is likely influenced by many genomic regions rather than a single locus, and therefore tends to exhibit a polygenic pattern of inheritance [69–70]. Flower filling distinguishes single from double-flowered varieties, with fully double forms being of high interest for carnation breeders. In rose, petal doubling is controlled by a single dominant gene on chromosome 3 [54, 71], while in diploid carnation the dominant allele of the D85 locus has been shown to be responsible for petal doubling [72]. Here, one SNP marker explaining 8.85% of phenotypic variation was identified for flower filling by the simplex dominance model, supporting a dominant gene action underlying this trait in tetraploid pot carnation. Sequence alignment of the flanking region of D85 against the reference genome used in this study could provide evidence of co-localisation between the previously reported and presently detected loci. Still, confirmation of a monogenic inheritance pattern for flower filling in carnation will require further studies across additional populations.

Ornamental crops are predominantly clonally propagated, with polyploid varieties being widely adopted in commercial production. These characteristics result in highly heterozygous fixed genotypes, in which nonlinear interactions, like dominance between alleles, may play a major role in shaping the genetic architecture of complex traits [73]. The modeling of dominance effects in association studies has been investigated in clonally propagated polyploid crops, including potato, blueberry and rose [23, 54, 74]. For these species, accounting for dominance has shown to improve statistical power for QTL detection in association analyses. In the present study, 18 of the 21 significant signals detected were identified by either the simplex or duplex dominance models. Moreover, for the seven traits where a significant signal was detected, at least one of these signals came from one of the dominance models. These results indicate that, for the loci detected here, dominance-based gene action models identified more significant associations than the additive model alone, highlighting the value of considering multiple gene action models in clonally propagated species, where genotypes remain fixed across generations. This reflects detection power within the present GWAS rather than a direct, generalizable estimate of the overall contribution of dominance to these traits.

Here, we carried out the first GWAS for eight key ornamental traits in pot carnation. Significant association signals were detected for most traits, and comparisons could be drawn to locus-trait analyses in other ornamental and crop species. However, QTL with minor effects may have remained undetected. Although the Meff significance threshold adjustment is less stringent than the standard Bonferroni adjustment, it may still lack the sensitivity required to identify minor QTL contributing to phenotypic variation, especially in more complex traits. Moreover, the final phenotype for complex traits is influenced by multiple underlying components, for example, the various parts and physiological mechanisms that contribute to plant height, which collectively determine its overall height. Association studies where the multiple variables that compose a trait are studied individually would further contribute to the dissection of the traits as a whole.

### Genomic prediction of ornamental traits in tetraploid carnation

Genomic prediction is widely regarded as an effective strategy for improving quantitative traits and accelerating overall genetic gain in breeding programs [4, 75]. In polyploid species, such as rapeseed oil, tobacco, sugar cane and potato, genomic selection has also demonstrated potential to increase genetic gain [27, 29, 76–77]. To obtain genetic marker effects from polyploids, the standard approach is to consider the size of the marker effect as proportional to the dosage of the minor allele. However, having multiple heterozygous states adds complexity to the genetic analysis of traits [15–16]. To circumvent this, one approach is to use diploidized genetic markers, where all heterozygous genotypes are collapsed into a single class [29], what we here referred to as pseudo-diploid. However, this comes at the cost of losing information about the different heterozygous states, which can be particularly unfavourable when dealing with clonally propagated species. Conversely, assuming each genotypic state has its own effect (e.g. in tetraploids each SNP marker would have five possible effects) can account for both additive and non-additive effects, which has been reported to improve prediction accuracy in autotetraploids [26]. Since allopolyploids largely behave as diploids in terms of chromosome pairing and inheritance, SNP calling and genotypic marker encoding are generally performed as in diploid organisms. Here we therefore draw comparisons primarily with autotetraploids and segmental allopolyploids.

In autotetraploid species, such as sugar cane and blueberry, the difference in prediction accuracy between diploidized and tetraploid dosage markers has been reported to be minimal. Despite these species having polysomic inheritance, the authors argue that the high frequency of homozygous markers and single-dosage alleles likely leads to a similar performance between diploidized and tetraploid dosage markers in genomic prediction [29, 73]. In tropical forage grass species (Urochloa spp.), described as segmental allopolyploids, genomic prediction for different gene action models has been investigated for several agronomic traits [28]. The authors evaluated genomic prediction accuracy based on allele dosage information, either diploidized or tetraploid dosage as well as additive only or additive and dominance gene action. Their results showed marginal difference in prediction accuracy between diploidized or tetraploid dosage marker data, and a slight advantage when considering additive effects only over additive and dominance effects. Here, we carried out genomic prediction based on three SNP marker encoding schemes, representing different allele dosage information and gene action models on a tetraploid carnation diversity panel. Prediction accuracy remained largely unchanged regardless of the SNP marker encoding scheme (Fig. 4). Simplifying the three heterozygous states into one had little effect on prediction accuracy. Similar to reports for sugar cane and blueberry, the frequency of homozygous markers is relatively large in our dataset, especially for the nulliplex class (homozygous for the reference allele), which represents around 50% of all SNP markers present (Additional file 1, Fig. S2). Regarding gene action, prediction accuracy was not affected when modeling additive and non-additive effects compared to additive effects only. Although fitting non-additive effects in genomic prediction models has reportedly improved prediction accuracy in several diploid species [78–79], similar to our results here, our previous study in diploid carnation reported no improvement in prediction accuracy when modelling both additive and non-additive effects together [12]. It is likely that our model is not accurately capturing and estimating non-additive effects. This is commonly observed when modeling dominance or epistatic effects in genomic prediction, where very large datasets or non-parametric models are needed to reliably capture their contribution [73, 80].

Incorporating QTL information into genomic prediction models can improve prediction accuracy in crop species [13, 47, 81]. Such information can be either historical, derived from previous studies and typically based on a marker dataset different from that used for genomic prediction, or newly detected, where the same dataset is used to perform both QTL mapping and genomic prediction. In rice, newly detected QTL were shown to further improve prediction accuracy compared to historical QTL information [81–82]. Leveraging newly detected QTL to improve prediction accuracy is additionally favourable in ornamental crops, for which historical QTL information is comparatively scarce [12]. In chrysanthemum, GWAS-assisted genomic prediction has been reported to improve prediction accuracy for flowering and plant architecture-related traits [10–11]. Similarly, in diploid cut carnation, the inclusion of top-scoring GWAS markers has been shown to improve prediction accuracy for vase life [12]. Here, we included top-scoring SNP markers detected by a GWAS to a RR-BLUP genomic prediction model to assess impact on prediction accuracy. Only three of the eight traits evaluated (earliness, flower filling, and branching) showed a marginal improvement in prediction accuracy over the baseline model (Additional file 1, Fig. S3). For the remaining traits, and for the AD encoding in general, fitting top-scoring SNP markers as fixed effects provided no benefit over the baseline model. This indicates that the top-scoring SNP markers detected by GWAS in this panel carry little additional predictive information beyond the genome-wide marker set already captured by the baseline model. As the best-performing marker count for each trait and encoding was itself selected by comparing prediction accuracy across configurations within the same repeated cross-validation design, these values should be interpreted as the best-performing numbers for this dataset and design, rather than as universally optimal marker counts. Given the small magnitude of the observed gains (at most 0.033), we do not consider a formal statistical evaluation, and interpret these gains as marginal rather than as reliable evidence of a genuine benefit. Two distinct factors limit the AD encoding here. First, at larger marker counts (n > = 100), the fixed effects matrix becomes too large relative to the number of training individuals to be estimated reliably, which is a dimensionality problem rather than evidence against non-additive effects. Second, even at the smaller, estimable marker counts shared with the PD and A encodings, the AD encoding did not outperform them, suggesting that modelling non-additive effects did not provide a genuine predictive benefit beyond the dimensionality issue. Nonlinear models such as neural networks or reproducing kernel Hilbert space regression with nonlinear kernels could prove more effective for leveraging non-additive effects in genomic prediction [83–84].

Although differences in prediction accuracy between the full tetraploid dosage encoding (A) and the diploidized encoding (PD) were minimal in this study (Fig. 4), allele dosage information remains biologically relevant for genetic analyses in tetraploid carnation. In carnation, polyploid commercial varieties have been developed through extensive intra- and interspecific hybridization between D. caryophyllus L. and related species including D. japonicus, D. chinensis, and D. barbatus [1, 21]. Furthermore, artificial chromosome doubling using antimitotic agents such as colchicine has been widely employed to generate polyploid breeding material and restore fertility in sterile interspecific hybrids [85]. Consequently, polyploidy across carnation cultivars has originated from different sources, which complicates their classification as either allo- or autopolyploid [86]. Moreover, a strictly binary classification does not accurately describe inheritance patterns in polyploids, as intermediate forms like segmental allopolyploidy or mixosomy are well recognized [18–19, 87]. Though characterising the inheritance mode of tetraploid carnation was beyond the scope of this study, we hypothesise that, like rose, the breeding history of modern carnation cultivars may be indicative of segmental allopolyploidy [20]. A definitive characterisation of the inheritance mode in tetraploid carnation would require formal quantification of preferential pairing, quadrivalent formation, and double reduction during parental meiosis [20].

Trait genetic architecture has been proposed as a key determinant of the optimal number of top-scoring SNP markers to include in a genomic prediction model [13, 47], with simulation and empirical studies in other species reporting improved prediction accuracy when fitting larger panels of GWAS-detected markers for polygenic traits [10, 47]. Here, gains in prediction accuracy were marginal and confined to a single or at most two top-scoring SNP markers for earliness, flower filling, and branching, and no configuration of top-scoring SNP markers improved prediction accuracy for the remaining five traits. Taken together, these results do not support a clear conclusion about the optimal number of top-scoring SNP markers for genomic prediction in this panel. Factors such as SNP marker density, population structure, and population size, which influence the genomic heritability of a trait, remain relevant to prediction accuracy in GWAS-assisted genomic prediction [88–89], and larger diversity panels may be needed to detect real benefit of this approach in tetraploid carnation.

## Conclusions

Our findings contribute to the better understanding of the genetic basis of key ornamental traits in carnation, as well as the potential of genomic selection for this ornamental species. All reported prediction accuracies were obtained from random splits within this diversity panel, and extension to distinct populations, later breeding generations, or prospective selection candidates remains to be evaluated. We identified genomic regions potentially associated with the traits under study and characterized their distribution across the genome. Beyond the standard additive model, the simplex and duplex dominance models highlighted the importance of accounting for non-additive effects in marker-trait association analyses in clonally propagated tetraploids. By contrast, accounting for non-additive effects in genomic prediction did not improve prediction accuracy. To better capture the nonlinear relationships from dominance or epistatic gene action, the implementation of nonparametric approaches such as deep learning may be beneficial, particularly in polyploid species, where this type of interaction is expected to be more complex [15, 73, 84].

## Supplementary Information

Below is the link to the electronic supplementary material.

Supplementary Material 1

## Data Availability

The datasets presented in this study can be found in the following online repository: [https://github.com/hhtaveras/tetraploid_carnation].

## Abbreviations

GWAS: Genome-wide association study
QTL: Quantitative trait locus
SNP: Single nucleotide polymorphism
BLUP: Best linear unbiased prediction
BLUE: Best linear unbiased estimate
RR-BLUP: Ridge regression best linear unbiased prediction
MAF: Minor allele frequency
PVE: Phenotypic variation explained
PCoA: Principal coordinate analysis
GBS: Genotyping-by-sequencing
PD: Pseudo-diploid encoding
A: Additive encoding
AD: Additive and non-additive encoding
