# Supplementary material for: Genome-wide association studies and genomic prediction for key ornamental traits in tetraploid pot carnation
Source: BMC Plant Biol. 2026 Oct 27;26:1927. doi: 10.1186/s12870-026-10279-1 (PMC13750050; doi:10.1186/s12870-026-10279-1)
Supplement: Supplementary file 1 [file 12870_2026_10279_MOESM1_ESM.pdf]

Genome-wide association studies and genomic prediction for key ornamental traits in tetraploid carnation

Hugo H. Tavera[1,2*], Dominik Losert[2], Maike Boxriker[2], Robert Boehm[2], Lydia Kienbaum[2], Dutta Sonali[2], Carola Zenke-Philippi[1]

[1*]Institute of Agronomy and Plant Breeding II, Justus Liebig University, Giessen, Germany.
[2]Klemm + Sohn GmbH & Co KG, Stuttgart, Germany.

*Corresponding author(s). E-mail(s): hugo.tavera@agrar.uni-giessen.de; Contributing authors: d.losert@selecta-one.com; m.boxriker@selecta-one.com; r.boehm@selecta-one.com; l.kienbaum@selecta-one.com; s.dutta@selecta-one.com; carola.zenke-philippi@uni-giessen.de;

Supplementary methods

Phenotypic trial summary

Table S1 Number of cultivars, check cultivars, plants per plot, traits scored, and total and missing observations, for each location-year combination

Loc.	Yr	Cv.	Check cv.	Check rep.	Plants/plot	Traits	Total obs.	Missing obs.
Stuttgart	2023	316	4	12	12 (6 checks)	11	4797	767
Stuttgart	2024	327	4	12	12 (6 checks)	11	4849	1204
Italy	2023	314	4	12	12 (6 checks)	11	4680	1075
Italy	2024	316	4	12	12 (6 checks)	13	4745	81

The second-stage multi-environment model described in Section 2.1 of the main text was fitted in R using the ‘lme4’ package (Bates et al. 2015) as follows.

```
lmer(y ~ 1 + (1|location) + (1|year) + (1|location:year) +
   (1|genotype) + (1|genotype:location) + (1|genotype:year),
   data = data)
```

Table S2 Heritability (H^2) and genotypic BLUP range for each trait, estimated from the two-stage mixed model described in Section 2.1, across all four trials

Trait	H^2	Min	Mean	Max
Earliness	0.64	148.40	177.00	197.58
Flower filling	0.89	1.28	4.35	7.91
Flower count	0.53	4.11	5.55	6.64
Flower size	0.76	2.74	5.70	7.60
Branching	0.26	4.56	4.96	5.44
Habit	0.65	2.81	5.28	7.60
Plant height	0.76	2.66	4.05	6.80
Vigour	0.74	3.01	4.80	7.39

Effective number of markers (M_{eff}) for the GWAS significance threshold

A significance threshold of $p < 0.05$ was set for each GWAS gene-action model using the M_{eff} adjustment implemented in the `set.threshold` function of 'GWASPoly' (`method = "M.eff"`), a Bonferroni-type correction using an effective number of markers estimated from pairwise marker correlations. Following Moskvina and Schmidt (2008, eq. 4 and 6), for each chromosome the effective number of tests is computed as

$$K_{\text{eff}} = 1 + \sum_{j=2}^{m} \kappa_j, \qquad \kappa_j \approx \sqrt{1 - r_j^{-1.31 \log_{10} \alpha}},$$

where r_j is the maximum pairwise correlation of the j-th marker (ordered by position) with any preceding marker on the same chromosome, m is the number of markers tested on that chromosome, and $\alpha = 0.05$ is the target significance level. The genome-wide M_{eff} is the sum of K_{eff} over all chromosomes, and the resulting significance threshold is $-\log_{10}(\alpha/M_{\text{eff}})$.

This correction was applied independently for each gene-action model parameterization tested by 'GWASPoly' (additive, 1-dom-alt, 1-dom-ref, 2-dom-alt, 2-dom-ref). The number of markers tested varies for each model after 'GWASPoly' checks whether markers are usable under each model encoding. For the dominance models specifically, each marker is recoded into a binary indicator (e.g. dosage ≥ 1 vs. dosage $= 0$ for 1-dom-alt), and if that particular binary split is too imbalanced, the marker is dropped for that model only, even if it passed the MAF check. Table S3 reports for each model the number of markers tested, the resulting effective number of markers, and the corresponding significance threshold.

Table S3 Number of markers tested, effective number of markers (M_{eff}), and resulting significance threshold, for each GWAS gene-action model

Model	Markers tested	M_{eff}	$-\log_{10}(p)$ threshold
Additive	15,433	10,943	5.34
1-dom-alt	15,298	10,842	5.34
1-dom-ref	14,203	10,077	5.30
2-dom-alt	15,318	10,849	5.34
2-dom-ref	15,027	10,628	5.33

Supplementary figures

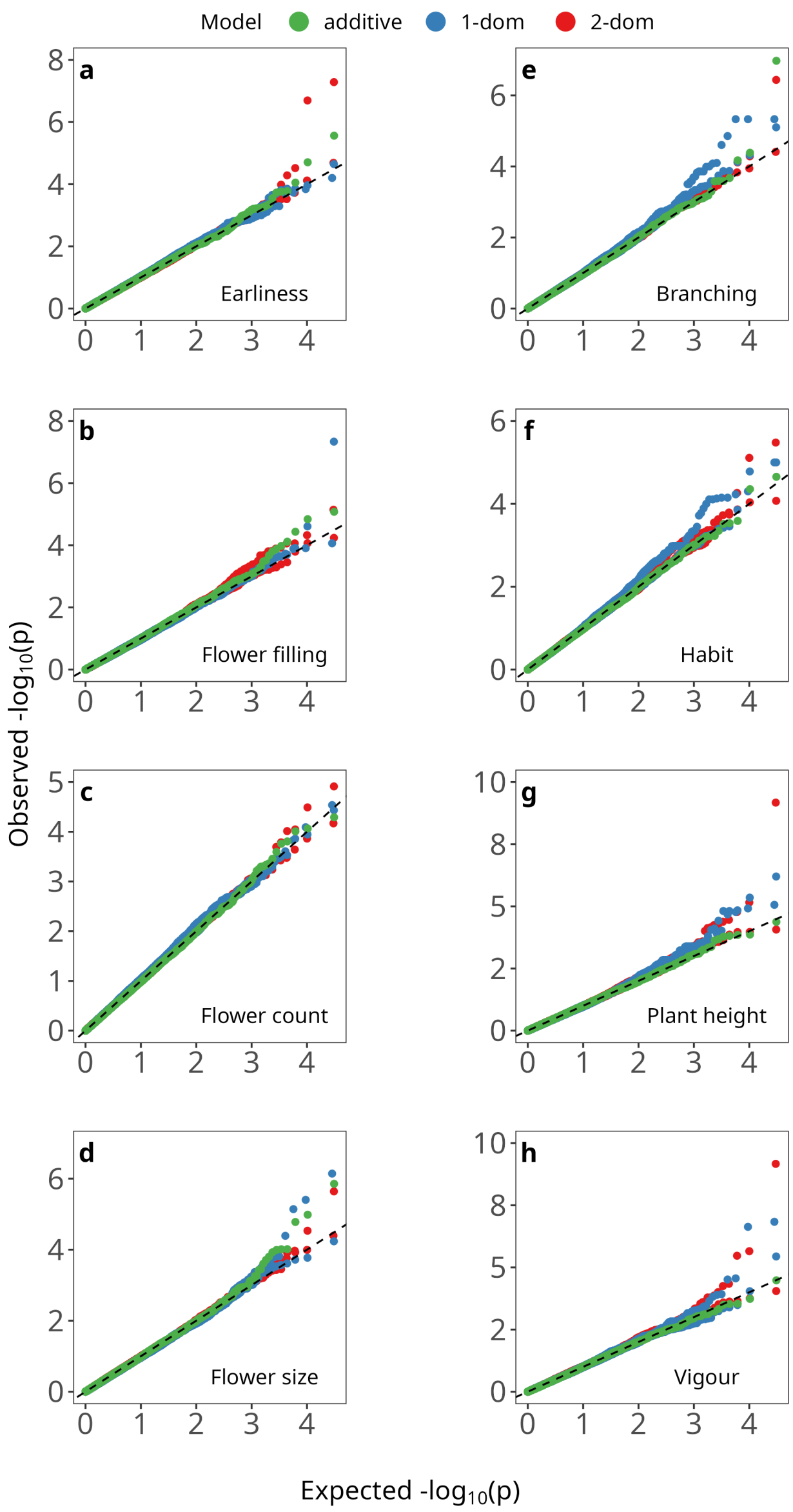

Fig. S1 QQ-Plots of GWAS for eight ornamental triats. Three gene action models were assessed for 287 cultivars and 15,433 SNP markers, additive (green), simplex dominance (blue), and duplex dominance (red). **a** Earliness. **b** Flower filling. **c** Flower count. **d** Flower size. **e** Branching. **f** Habit. **g** Plant height **h** Vigour.

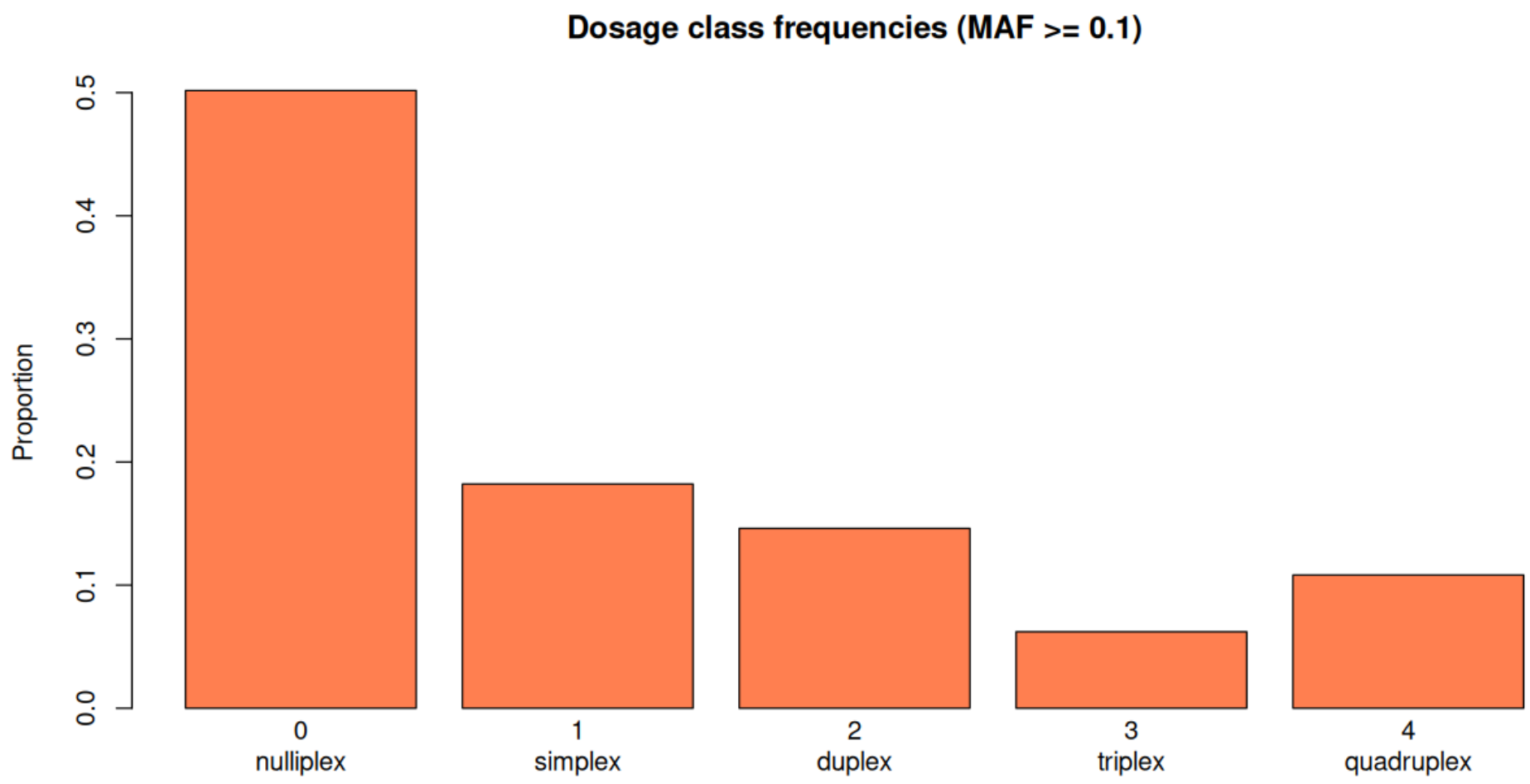

Fig. S2 Proportion dosage classes in the SNP marker dataset

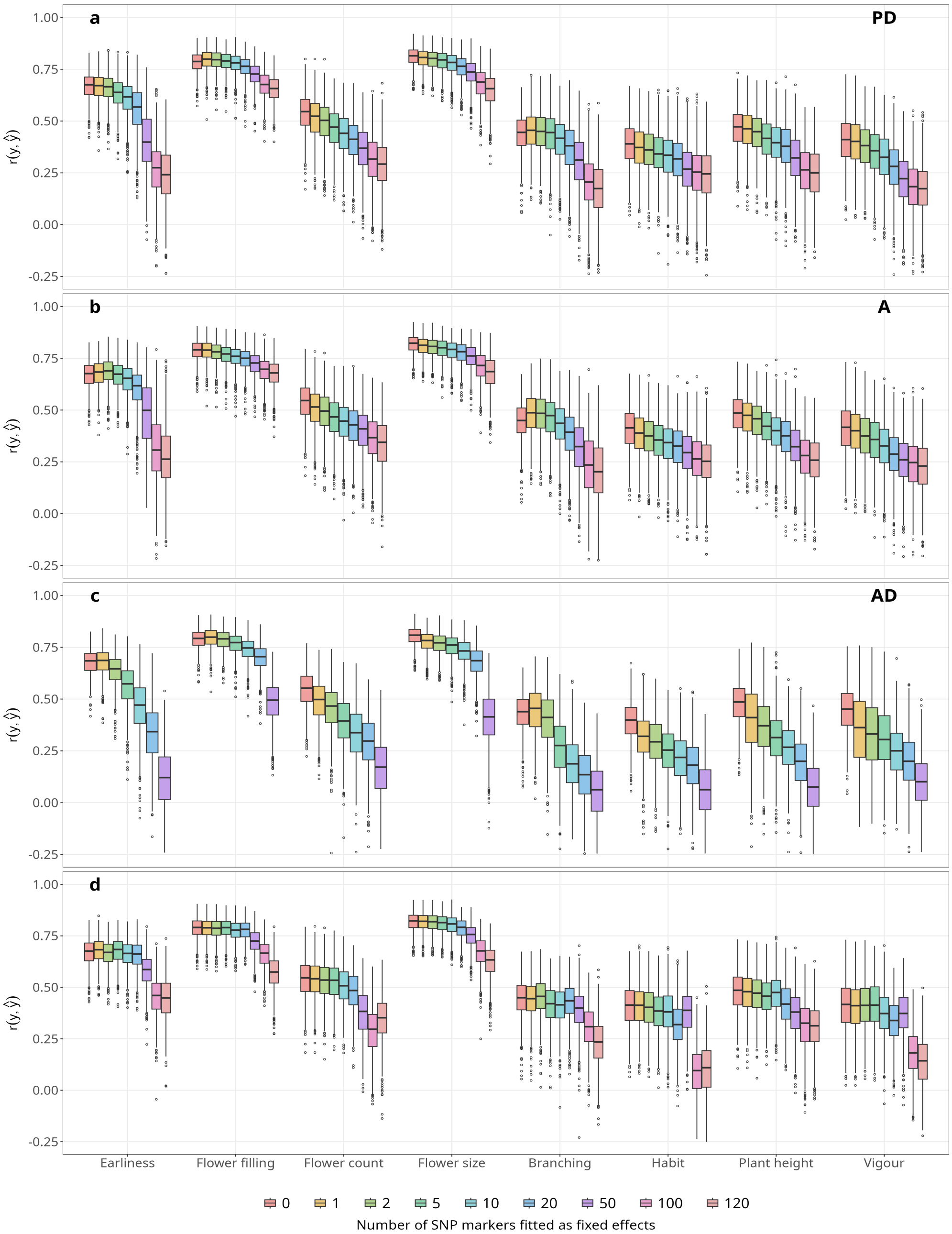

Fig. S3 Genomic prediction on a tetraploid carnation diversity panel for eight ornamental traits fitting 0 to 120 top-scoring GWAS markers (**a** – **c**) or 0 to 120 random SNP markers (**d**) as fixed effects, with marker selection nested within each cross-validation fold. **a** Pseudo-diploid (PD) encoding. **b** Additive only (A) encoding. **c** Additive and non-additive (AD) encoding. **d** Additive only (A) encoding fitting random SNP markers. The y-axis is fixed from -0.25 to 1 for all panels; outlier values below -0.25 are not shown

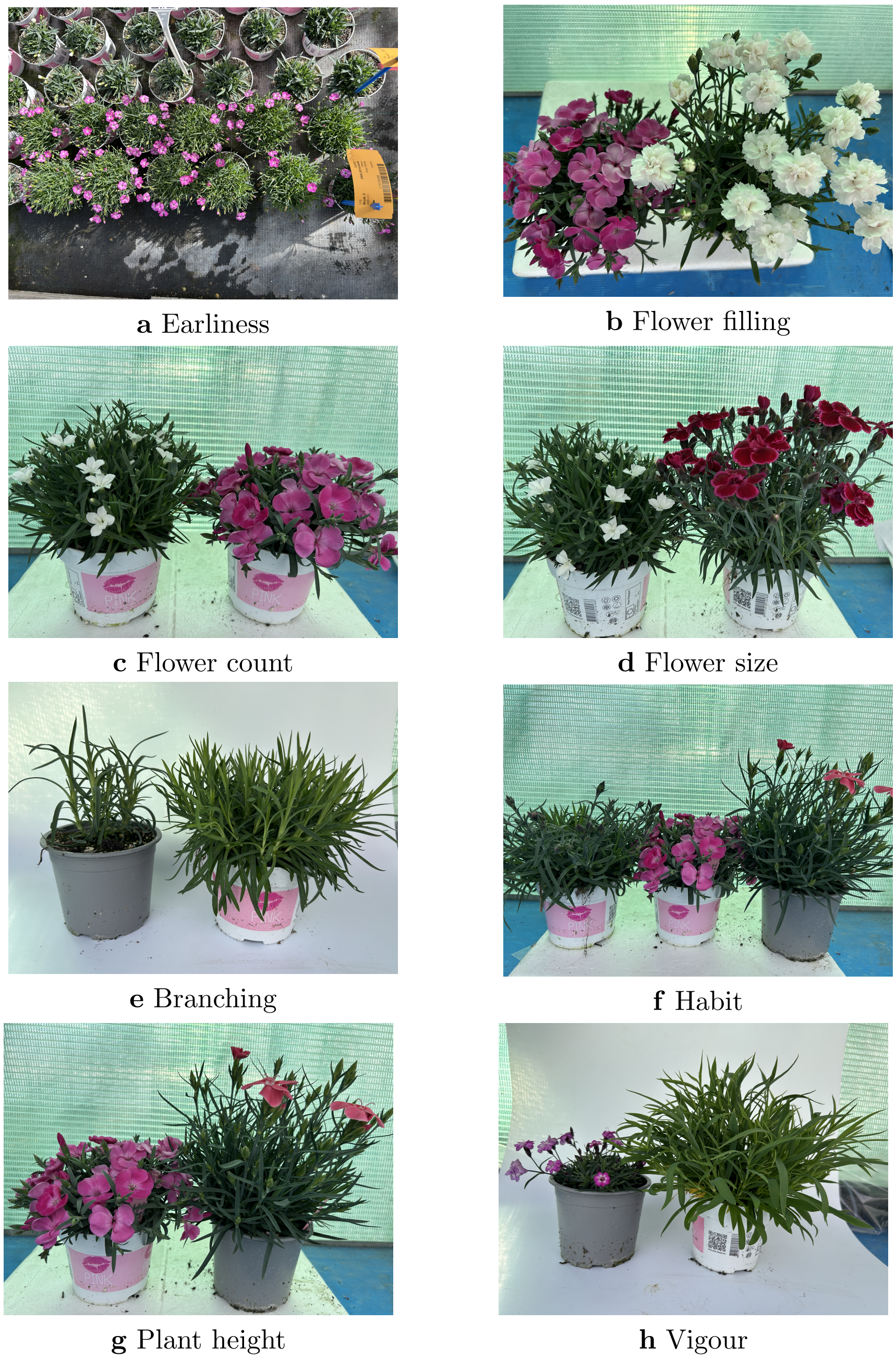

Fig. S4 Reference photographs used to guide field scoring of the eight ornamental traits. The plants shown illustrate the range of phenotypic variation within each trait rather than the precise developmental stage used for scoring in the trial. **a** Earliness. **b** Flower filling. **c** Flower count. **d** Flower size. **e** Branching. **f** Habit. **g** Plant height. **h** Vigour
